# Cytological and molecular characterization of three gametoclones of *Citrus clementina*

**DOI:** 10.1186/1471-2229-13-129

**Published:** 2013-09-10

**Authors:** Maria Antonietta Germana, Pablo Aleza, Esther Carrera, Chunxian Chen, Benedetta Chiancone, Gilles Costantino, Dominique Dambier, Xiuxin Deng, Claire T Federici, Yann Froelicher, Wenwu Guo, Victoria Ibáñez, José Juárez, Kevin Kwok, François Luro, Marcos A Machado, Miguel Angel Naranjo, Luis Navarro, Patrick Ollitrault, Gabino Ríos, Mikeal L Roose, Manuel Talon, Qiang Xu, Fred G Gmitter

**Affiliations:** 1 Università degli Studi di Palermo, Dipartimento di Scienze Agrarie e Forestali, Viale delle Scienze, 11, Palermo 90128, Italy; 2 IVIA, Centro de Genómica, Moncada, Valencia, Spain; 3 University of Florida, Citrus Research and Education Center, Lake Alfred, FL, USA; 4 CIRAD, Département “Systèmes Biologiques” Unité de Recherche ‘Multiplication Végétative’ Montpellier, Paris, France; 5 Huazhong Agricultural University, Wuhan, Hubei, China; 6 University of California, Department of Botany and Plant Sciences, Riverside, CA, USA; 7 INRA, UR GEQA, San Giuliano, France; 8 Instituto Agronômico de Campinas, Centro APTA Citros Sylvio Moreira, Cordeirópolis, SP, Brazil; 9 IVIA, Centro de Proteccion Vegetal y Biotecnologia, Moncada, Valencia, Spain; 10 USDA-ARS, Southeastern Fruit and Tree Nut Research Laboratory, Byron, GA, USA

**Keywords:** Anther culture, Gynogenesis, Gametoclonal variation, Genome sequencing

## Abstract

**Background:**

Three gametoclonal plants of *Citrus clementina* Hort. ex Tan., cv. Nules, designated ESP, FRA, and ITA (derived from three labs in Spain, France, and Italy, respectively), were selected for cytological and molecular characterization in order to elucidate genomic rearrangements provoked by haploidization. The study included comparisons of their ploidy, homozygosity, genome integrity, and gene dosage, using chromosome counting, flow cytometry, SSR marker genotyping, and array-Comparative Genomic Hybridization (array-CGH).

**Results:**

Chromosome counting and flow cytometry revealed that ESP and FRA were haploid, but ITA was tri-haploid. Homozygous patterns, represented by a single peak (allele), were observed among the three plants at almost all SSR loci distributed across the entire diploid donor genome. Those few loci with extra peaks visualized as output from automated sequencing runs, generally low or ambiguous, might result from amplicons of paralogous members at the locus, non-specific sites, or unexpected recombinant alleles. No new alleles were found, suggesting the genomes remained stable and intact during gametogenesis and regeneration. The integrity of the haploid genome also was supported by array-CGH studies, in which genomic profiles were comparable to the diploid control.

**Conclusions:**

The presence of few gene hybridization abnormalities, corroborated by gene dosage measurements, were hypothetically due to the segregation of hemizygous alleles and minor genomic rearrangements occurring during the haploidization procedure. In conclusion, these plants that are valuable genetic and breeding materials contain completely homozygous and essentially intact genomes.

## Background

Haploid plants or their derivatives, e.g. doubled haploid (DH) or tri-haploid (TH), are valuable in conventional breeding and genetic studies. However, most *Citrus* genomes are highly heterozygous, and it is practically impossible to develop homozygous lines through conventional hybridization, due to sexual incompatibility, nucellar embryony, severe inbreeding depression, and long juvenility. Gametic embryogenesis is a single-step approach to produce homozygous clones from heterozygous parents [1-7], from which most *Citrus* haploids were generated.

*In situ* parthenogenesis induced by irradiated pollen, followed by *in vitro* embryo culture has been reported in *Citrus*[8-11]. Haploid plantlets have been recovered by anther culture from *Poncirus trifoliata* L. Raf. [12] and *C. madurensis* Lour. [13]. A doubled haploid plantlet has been obtained from the hybrid No. 14 of *C. ichangensis* × *C. reticulata*[14]. Haploid plantlets and highly embryogenic haploid calli were recovered from *C. clementina* Hort. ex Tan. [15-17]. Haploid, but albino embryos, arose from cultures of ‘Mapo’ tangelo (*C. deliciosa* × *C. paradisi*) [18]. In other reports, haploid and diploid calli, embryos and leafy structures, but no green plants, were produced from culture of *C. limon* L. Burm. f. anthers [19]. Haploid embryos of *Clausena excavata*[20] and homozygous short-lived plantlets of Rhode Red Valencia sweet orange [21] have also been reported.

The objective of this work was to elucidate the effect of haploidization in the genome structure of three different gametoclonal plants of *Citrus clementina* Hort. ex Tan., cv. Nules. To compare their genomes, the three gametoclones obtained by gynogenesis or by pollen embryogenesis, were freely provided by research groups in Spain (Navarro), France (Ollitrault), and Italy (Germanà). The tissues and DNA samples from the three candidate plants have been analyzed and characterized using various technologies and methods by laboratories in several institutions worldwide to assure that they are free of large deletions or other defects, as well as to confirm their homozygosity (mono-allelic at any locus analyzed). Specifically, candidate tree chromosome numbers were verified for their ploidy levels. The candidate genomes were evaluated using genomic or EST-derived SSR markers and microarray technology. The collaborative results on the three materials are reported here in detail.

## Methods

### Plant material

Three gametoclonal plants, designated ESP, FRA, and ITA respectively acquired in the lab of Navarro (Spain), Ollitrault (France), and Germanà (Italy), were all derived from *Citrus clementina* Hort. ex Tan., cv. Nules and preliminarily shown to be homozygous based on some selected loci. They were obtained by *in situ* parthenogenesis induced by irradiated pollen followed by *in vitro* embryo culture, or by pollen embryogenesis. Specifically, ESP was through *in vivo*-induced gynogenesis by pollination of Nules Clementine with irradiated pollen of Fortune mandarin followed by *in vitro* embryo rescue [22], FRA also through gynogenesis by pollination in the field with irradiated Meyer lemon (*Citrus meyeri* Y. Tan.) pollen [11], and ITA was obtained through anther culture of *C. clementina* cv. Nules [15,17]. ESP was previously characterized as a haploid [22]. All three plants were much less vigorous than the heterozygous mother plant, as revealed by leaf size and growth habit (Additional file 1: Figure S1). Samples from all three plants were sent to the respective laboratories of the collaborators for the specific analyses to which each group had committed.

### Chromosome number determination

Root- and shoot-tip chromosome counting was conducted using DAPI (4,6-diamidino-2-phenylindole) and hematoxylin staining techniques [23], respectively.

### SSR genotyping and analysis

SSR markers presumably or evidently heterozygous in the diploid Clementine control were selected for genotyping and analysis to determine if the three plant genomes were homozygous and complete at the various loci examined. Five laboratories were involved (Table 1), and primer sets, amplification conditions, and separation methods were summarized. At CIRAD, INRA, and IVIA, amplifications were performed according to Froelicher *et al.*[24] in a thermocycler (PTC-200, MJ Research) using 10 ng of citrus DNA, 0.2 μM of each primer and 0.8 unit of *Taq* polymerase (Goldstar, Eurogentec). The annealing temperature for all primers was 55°C. The 39 EST-SSR primers used in this study were selected based on their representation of each of the linkage groups defined in a Clementine genetic map [25,26]. At the University of Florida - CREC, 40 EST-SSR markers, likewise well distributed among the linkage groups in the diploid Clementine genetic map [25], were chosen for genotyping. All the genotyping, computing, and scoring procedures were previously described in detail [27,28]; similar methods were used at CCSM [29]. At the University of California, Riverside (UCR) amplifications were performed essentially according to Barkley *et al.*[30], except that PCR products were labeled by adding a 19 or 20-base M13 tail to the 5′ end of one sequence-specific primer and including an M13F or M13R primer carrying a dye label (LiCor IRD700 or IRD800) in each PCR reaction [31].

**Table 1:** Summary of SSR marker analysis of haploids from Clementine

**No. of PCR products in diploid clementine**	**No. of PCR products in ITA, ESP, and FRA**	**CREC**	**INRA**	**CCSM**	**CIRAD/IVIA**	**UCR**	**Total**
1	1	0	0	0	0	45	45
1	0 or 1	0	0	0	0	2	2
2	1	40	39	9	41	57	186
2	1 or 2	0	0	1	0	3	4
Total markers		40	39	10	41	107	237
Anomalous		0	0	1	0	5	6

### Array-CGH analysis

Array-CGH was performed as described in Rios *et al*. [32]. Genomic DNA was isolated from leaves [33]. Four Cy3- or Cy5-labelled samples from each gametoclonal plant were co-profiled on four 20 K *Citrus* cDNA microarrays containing 21240 EST, using Cy5- or Cy3-labelled control genomic DNA, respectively [34]. To prepare labelled probes, Cy3- or Cy5-dCTP fluorescent nucleotides (Amersham Biosciences) were incorporated directly in control and gametoclonal genomic DNA (2 μg) using BioPrime Array CGH Genomic Labelling System (Invitrogen). Each pair of purified Cy5 and Cy3 probes (about 50 μl each) was combined and mixed with 30 μg Cot-1 DNA (Invitrogen), 100 μg yeast tRNA (Invitrogen), and 346 μl TE buffer pH 7.4. Samples were concentrated with a microcon YM-30 filter (Millipore), and SSC buffer and SDS were added to reach a final volume of 60 μl containing 3.4× SSC and 0.3% SDS. For microarray hybridization, the probe mixture was denatured by heating at 97°C for 5 minutes, and immediately incubated at 37°C during 30 minutes to block repetitive DNA sequences. Hybridization mixture was applied to a 37°C pre-warmed hybrid-slip (Sigma), and a pre-warmed array slide was lowered onto the mix. Microarrays were hybridized in darkness at 65°C overnight (16–20 hours) using a glass array cassette following manufacturer’s instructions (Ambion). To prevent evaporation of hybridization solution during incubation, 5 μl of 3× SCC were poured into the reservoir inside the cassette chamber. Following hybridization, microarray slides were placed in a rack and the cover slip removed by 10 minutes immersion in a washing chamber containing 2× SSC and 0.03% SDS at room temperature (RT). Microarray slides were passed through a series of washes on a shaking platform. Wash series were as follow: 2× SSC, 0.03% SDS for 5 min at 65°C, followed by 1× SSC for 5 min at RT, and 3× 15 min washes in 0.2× SSC at RT. Microarray slides were dried by centrifugation for 5 min at 300 rpm. Arrays were immediately scanned at 5 μm. Cy3 and Cy5 fluorescence intensity was collected by using a ScanArray Gx (Perkin Elmer). The resulting images were overlaid and spots identified by the ScanArray Express program (Perkin Elmer). Spot quality was first measured by the signal-to-background method with parameters lower limit (200) and multiplier (2), and subsequently confirmed by visual test. The results were normalized for labeling and detection efficiencies of the two fluorescence dyes, prior to determining differential gene expression between haploid and diploid citrus samples. Intensities of selected spots were transformed into log2 (Cy3/Cy5) and data were normalized by the locally weighted linear regression (LOWESS) method. Genespring vs 7.3 software (Agilent, http.//http://www.agilent.com) was used to normalize values for each gene and for data analysis. Differentially regulated genes were ranked on the basis of signal intensity, normalized ratio, flag value and variance across 4 replicate experiments. Filtered genes identified to be differentially expressed by haploid/diploid signals lower than 0.3 with a P-value not higher than 0.05 were considered for subsequent gene dosage measurements. One-way ANOVA, parametric test without the assumption of equal variances was used to define differentially expressed genes.

### Gene dosage measurement

Quantitative real-time PCR was performed on a LightCycler 2.0 instrument (Roche), using the LightCycler FastStart DNA MasterPLUS SYBR Green I kit (Roche). Reaction composition and conditions followed manufacturer’s instructions. Each individual PCR reaction contained 2 ng of genomic DNA from gametoclonal or diploid control [33]. Cycling protocol consisted of 10 min at 95°C for pre-incubation, then 40 cycles of 10 sec at 95°C for denaturation, 10 sec at 60°C for annealing and 10–25 sec at 72°C for extension. Fluorescent intensity data were acquired during the extension time. Specificity of the PCR reaction was assessed by the presence of a single peak in the dissociation curve after the amplification and through size estimation of the amplified product. For gene dosage measurements, the relative quantification-monocolor analysis from the LightCycler Software 4.0 package (Roche) was used. This program compares the ratio of a target sequence to a reference DNA sequence, i.e. the sequence in the gametoclonal sample with the sequence in a diploid wild type sample. PCR and normalized calculations were repeated in at least three independent samples from each genotype, rendering an averaged estimation (± standard deviation) of target gene dosage in the haploids. Primers for the reference sequence are provided in Table 2.

**Table 2:** Primer sequences for each gene

**EST name**	**Primer sequence**
C04035D02	3′-CCCAAGCCAGATTTGATCAAGGGTC-5′
	3′-TGGATGTCACACCACTCCAGCAGAT-5′
IC0AAA36DF07	3′-AGCGCTCTTAAATCAACCCGTC-5′
	3′-GGATACTGCTGACTGATGTTGC-5′
IC0AAA34BC06	3′-TGATTCTCGTTTGAGGGTCCTC-5′
	3′-GCAATTCGCCACTTCAGGGTAA-5′
C34004E09	3′-ATGAAGTGTGAGGGTTGCGTTG-5′
	3′-TTCGGTCATGGTCTTCAGAGGT-5′
IC0AAA74CE10	3′-CGGTTCAAGAGAGGAGTTGTTG-5′
	3′-GATGCAACACATCAGGTGGGAT-5′
C06013D07	3′-TGGATTTGCTTGGTGCACACTG-5′
	3′-GCTGTTTTCTTCAACTACAGATCC-5′
C08012E04	3′-AGTGGGATTTGGTGTGGCAAAC-5′
	3′-ACCTACTGGAAATCTGAAGACC-5′
IC0AAA56DH07	3′-CCTTCCTCATCCACTTTTCAGG-5′
	3′-CTGAGACAGAAGCGCAAACTTG-5′

C04035D02 was used as reference gene.

## Results and discussion

### Chromosome counts

**Figure 1:**
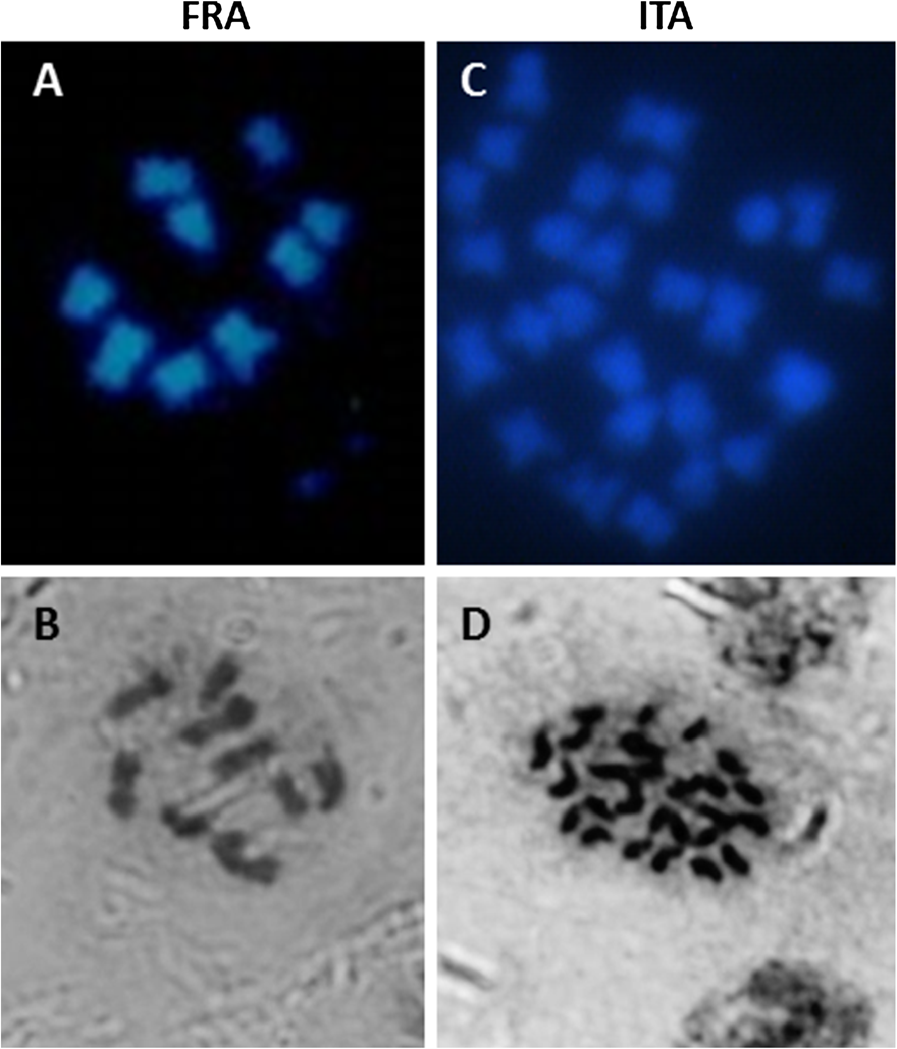
**Determination of chromosome number by DAPI (A, C) and hematoxylin (B, D) staining of chromosomes showing counts of 9 in the haploid plants from France (FRA) and 27 in the tri-haploid plant from Italy (ITA).** Chromosome count for the plant from Spain (ESP) was described in Aleza *et al.*[22].

After DAPI and hematoxylin staining of chromosomes in independent labs, 9 were found in ESP and FRA, and 27 chromosomes in ITA (Figure 1), confirming their haploidy and tri-haploidy, respectively. They were further confirmed by flow cytometry (Additional file 1: Figure S2).

### SSR marker analysis

A total of 237 SSR markers were selected, in many cases from previous mapping exercises, to represent as broad and unbiased coverage of the citrus genome as possible, and plant materials were genotyped (Table 1). No SSR alleles were detected in the gametoclones that were not present in diploid Clementine. At 232 loci the three gametoclones had one SSR allele also found in diploid Clementine. The gametoclones had the same allele as diploid Clementine at 45 of the 47 loci tested at which Clementine appeared homozygous. At two “anomalous” loci, the Clementine allele was observed in one or more of the gametoclones, and no allele was observed in the others. These two loci segregated for a null (no amplification) allele in a Clementine hybrid population, so these markers are also consistent with all gametoclones having complete, homozygous genomes. The gametoclones contained one of the two Clementine alleles at 183 of the 187 loci that were heterozygous in diploid Clementine. For three loci, the same two PCR products amplified from diploid Clementine were also observed in one or more of the gametoclones. Segregation of one of these loci was studied in a Clementine hybrid population and it was shown that these two PCR products segregated as a single Mendelian unit. This pattern could be caused by a tandem duplication of the amplified region, or by annealing of one PCR primer to nearly adjacent sites in the template DNA. Segregation of the other two loci has not been examined, but they could be explained possibly by a similar mechanism. Only one SSR locus revealed anomalous results in FRA, while the remainder revealed only a single allele product at all other loci surveyed.

### Array-CGH experiment

In order to detect putative genomic deficiencies, genomic DNA from ITA, ESP and FRA genotypes and the diploid wild type was labelled and hybridized to a 20 K citrus cDNA microarray [34] by array-CGH, a procedure that previously proved to be useful for the structural prediction of large genomic deletions in *Citrus clementina*[32]. Those ESTs showing a haploid/diploid signal ratio of 0.3 or lower with a maximum P-value of 0.05 for any of the genotypes were selected for further analysis. From 13 ESTs fulfilling these conditions, two of them were annotated as putative Cu/Zn-superoxide dismutase copper chaperones, which are the 5′end (C34004E09) and 3′end (KN0AAA2CB01) of the same citrus cDNA (Table 3). Under-represented ESTs were found in the three gametoclonal genotypes in a number ranging from 4 in ESP to 8 in ITA with 4 of them jointly found in two different genotypes.

**Table 3:** Underrepresented ESTs after array-CGH of haploid genomic DNA

**EST**	**Accession**	**Genotype ITA**		**Genotype FRA**		**Genotype ESP**		**Description**
		Normalized ratio (*)	P-value (**)	Normalized ratio (*)	P-value (**)	Normalized ratio (*)	P-value (**)	
IC0AAA36DF07	DY274520	2.08(0.98–3.45)	--	**0.04(0.03–0.06)**	1.8E-04	**0.04(0.01–0.09**)	5.0E-02	No annotation available
IC0AAA34BC06	DY273568	0.34(0.26–0.63)	1.4E-02	**0.24(0.17–0.31)**	1.5E-03	0.31(0.28–0.40)	1.0E-02	No annotation available
C34004E09	FC930172	0.95(0.87–1.05)	--	**0.13(0.09–0.20)**	1.7E-03	0.98(0.92–1.05)	--	Cu/Zn-superoxide dismutase copper chaperone
KN0AAA2CB01	DY256847	1.00(0.64–1.92)	--	**0.07(0.01–0.35)**	3.4E-02	0.78(0.39–2.19)	--	Cu/Zn-superoxide dismutase copper chaperone
C31204A07	FC874254	1.04(0.85–1.20)	--	**0.21(0.10–0.40)**	1.2E-02	2.33(1.78–3.87)	--	Thaumatin-like protein isoform 3
IC0AAA74CE10	DY290159	**0.22(0.18–0.30)**	1.3E-03	1.65(1.30–2.07)	1.4E-02	**0.21(0.18–0.27)**	6.3E-03	Nematode resistance-like protein
KN0AAK2BA02	DY259689	**0.14(0.07–0.19)**	3.6E-03	1.74(1.24–2.59)	3.9E-02	**0.21(0.14–0.26)**	1.4E-02	No annotation available
C06013D07	CX298593	**0.23(0.11–0.40)**	1.1E-02	1.26(1.11–1.42)	3.5E-02	1.37(1.29–1.45)	1.1E-02	No annotation available
C31810G10	FC924855	**0.18(0.10–0.50)**	1.7E-02	1.81(1.35–2.64)	3.8E-02	1.35(0.89–2.68)	--	Copine I-like protein
C08012E04	CX301690	**0.28(0.17–0.54)**	1.8E-02	1.87(1.40–2.58)	2.3E-02	1.28(0.83–2.99)	--	No annotation available
IC0AAA56DH07	DY282523	**0.30(0.12–0.63)**	4.0E-02	1.66(1.43–2.13)	1.2E-02	1.72(1.17–2.13)	--	No annotation available
IC0AAA89CG08	DY295961	**0.27(0.14–0.45)**	1.6E-02	0.43(0.28–0.99)	--	1.52(0.87–2.42)	--	No annotation available
KN0AAB2CE09	DY258014	**0.07(0.03–0.14)**	7.0E-03	1.59(1.16–2.14)	4.0E-02	**0.21(0.11–0.66)**	--	SJCHGC09076 protein

(*) Normalized haploid to diploid signal ratio. In bold, signal ratios lower than 0.3. Replicates with lowest and highest values are shown in brackets.

(**) Only Pvalues lower or equal to 0.05 are shown.

### Gene dosage experiment

**Figure 2:**
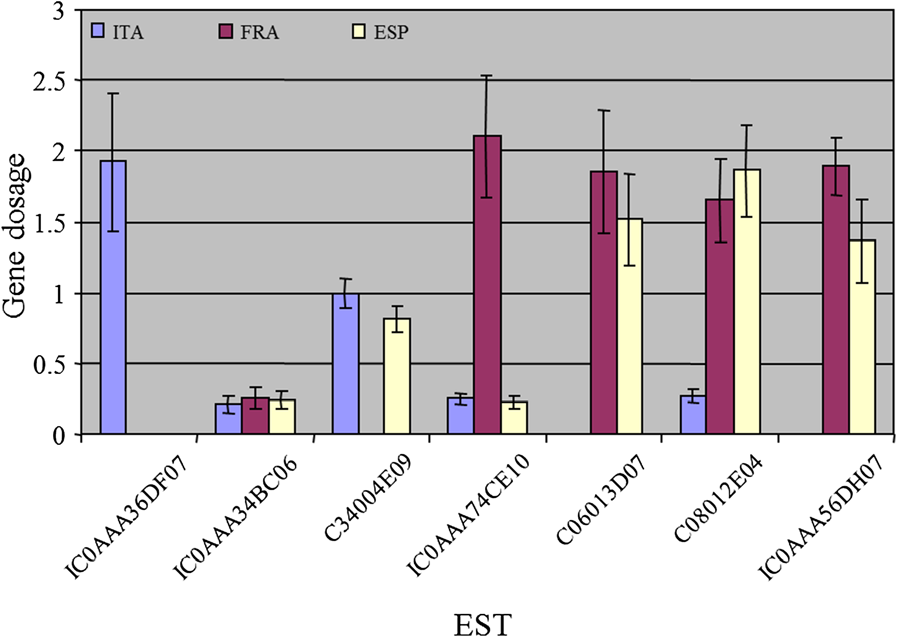
**Relative gene dosage of selected ESTs in the three haploids by real-time PCR.** Values are average and standard deviation of three determinations. Gene dosage is relative to the value of the diploid variety.

Seven of these ESTs were chosen for gene dosage evaluation by real-time PCR. Gene dosage measurements performed in this way confirmed the hybridization data presented in Table 3, except in two cases (Figure 2). Real-time PCR quantification failed to amplify ESTs C06013D07 and IC0AAA56DH07 that exhibited, respectively, microarray hybridization signal ratios of 0.23 and 0.30 in ITA, indicating that microarray data of these two ESTs was most likely affected by nonspecific cross-hybridization.

**Figure 3:**
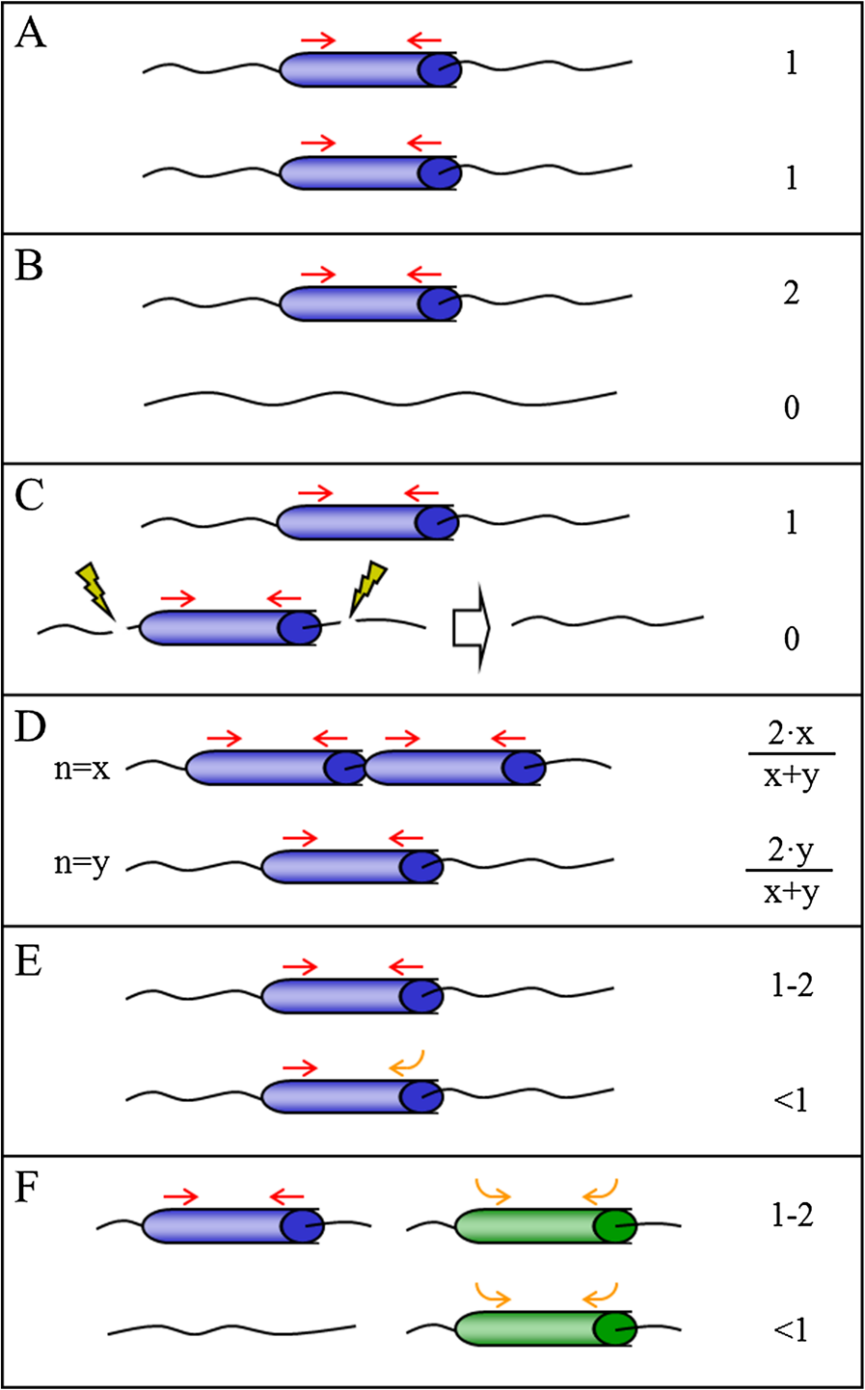
**Proposed models for the genomic mechanisms resulting in lower gene representation in the gametoclones.** Genes are depicted as blue and green cylinders and primers as red (specific) and orange (non-specific) arrows. Yellow flashes label deletion limits and the white arrow indicates conversion of standard to null allele. In each panel up and down drawings represent two alleles of a given locus in the diploid variety. The number or equation on the right is the relative gene dosage estimated for a haploid line inheriting the corresponding allele located on the same level. Gene dosages are always one for the diploid line. For further explanations see the text. **(A)***Standard case;***(B)***Hemizygotic model*; **(C)***Technical deletion model*; **(D)***Polymorphic tandem repeats model*; **(E)***Polymorphic sequence model*; **(F)***Hemizygotic model and cross-reaction*.

Since a gene dosage around one is expected for those genes that are neither enriched nor reduced upon haploidization, the estimation of gene dosages lower and higher than one in certain ESTs argues for the occurrence of genomic rearrangements during the haploidization procedure, or alternatively the segregation of hemizygotic genes, that are present exclusively in one of the two alleles. A schematic overview of these and other genomic explanations for the observed deviations in gene dosage is presented in Figure 3. In this Figure, the standard case of genes not affected by the haploidization procedure, with both haploids having identical alleles and therefore a constant gene dosage around one is exemplified in panel A. In the hemizygotic model, a gene or DNA fragment is absent in one of the alleles leading to gene dosages equal to zero in haploids carrying the deleted allele. In the diploid parental, the PCR amplification of hemizygotic genes is originated from only one allele, as the second allele is absent (Figure 3B). The relative enrichment of the gene in haploid individuals inheriting the full allele causes a two-fold increase in gene dosage, whereas haploids carrying the null allele have a value close to zero. In this work, IC0AAA36DF07, C06013D07 and IC0AAA56DH07 showed such a hemizygous-like behavior. In the deletion model, as outlined in Figure 3C, the gene is deleted during haploidization procedures producing a null allele that is not present in the diploid. EST C34004E09, for example, was not detectable in FRA but its content in ITA and ESP individuals was close to one. These data could be explained by a genomic deletion mechanism occurring during the haploidization process. Under this model, haploids losing the gene during the haploidization do not show PCR amplification signal, but those haploids inheriting an intact allele show a relative gene dosage similar to the original diploid. Finally, the remaining three ESTs analyzed by real-time PCR show low gene dosage values higher than zero in the three haploids (IC0AAA34BC06) or in at least one of them (IC0AAA74CE10 and C08012E04). Three different structural models were postulated to explain these observations. In the polymorphic tandem repeats model (Figure 3D), a tandemly-repeated gene found respectively ‘x’ and ‘y’ times in the two alleles, show a gene dosage value responding to the equations (2∙×)/(x + y) and (2∙y)/(x + y) in the two alternative haploids. Thus, a 5-fold ratio in the number of repeats would produce relative gene dosages of 10/6 and 2/6 in the resultant haploids, certainly similar to the observed values. Alternatively, polymorphic variations in the primer binding sequence on the gene might cause allele-specific modifications of PCR efficiency leading to variable gene dosages (Figure 3E, polymorphic sequence model). Another source of mis-estimation of gene dosage might result from the combination of hemizygosis and non-specific cross-reaction of the primers, as presented in Figure 3F that originate altered determinations of gene dosage in the resulting haploid genotypes. Thus, the results confirm that ITA, ESP and FRA genomes do not carry important fragment deletions or rearrangements, and the few genomic differences observed between the diploid and haploid genotypes can be explained to a large extent by the natural heterozygosity of the diploid parental.

## Conclusion

In this study, chromosome counting confirmed that ESP and FRA were haploid and ITA tri-haploid. Among a total of 237 SSR markers, most were selected from previous mapping exercises and represented broad and unbiased coverage of the citrus genome. 231 markers detected a single allele in ITA, ESP and FRA; each allele also existed in the diploid Clementine genome. Of the six SSR loci with anomalous results, segregation in Clementine was studied for three loci and in these cases the anomalous results in the haploids were shown to be caused by similar anomalies in Clementine. The array-CGH experiment revealed that only 13 cDNAs had anomalous results among more than 20,000 cDNAs on the array. After real-time PCR of 7 of these genes, only four showed a gene dosage close to zero in one or two candidates, so no relevant gene loss was detected in any of the three genomes. Consequently array-CGH, in addition to all other characterization methods employed, provided compelling evidence that haploidization of citrus through *in situ* parthenogenesis induced by irradiated pollen followed by *in vitro* embryo culture, or by pollen embryogenesis, does not generate substantial genome rearrangements. Therefore, these three gametoclones can be used, with no concerns regarding their genomic integrity, for genetic studies as well as for citrus improvement, for example, through di-haploidization. In addition, it is noteworthy that the conclusions reached in this study, that haploidization does not disrupt the natural citrus genome structure, provided the major basis for the selection of the target citrus genome for producing the reference sequence for the international citrus research community [22].

## Competing interests

The authors declare that they have no competing interests.

## Authors’ contributions

MAG provided one of the gametoclones (ITA), contributed to the design of the project, and wrote the first draft of the manuscript. PA provided one of the gametoclones (ESP), and contributed to its characterization. EC extracted DNA and analyzed microarray data. CC performed the SSR analysis at UF-CREC and contributed to the design of the project, and to writing and revision of the manuscript. BC contributed to the development and characterization of one of the gametoclones (ITA). GC contributed to the SSR genotyping at INRA. DD performed chromosome counts and flow cytometry at CIRAD. XD, WG and QX performed chromosome counts at HAU. CF, KK, and MR performed SSR genotyping at UCR; MR contributed to the design of the project and manuscript revisions. JJ contributed to the characterization of ESP gametoclone. FL contributed to the SSR genotyping at INRA, and provided genetic linkage maps of diploid Clementine. MM contributed to SSR genotyping at CCSM, and to the design of the project. VI prepared plant material and was involved in revising the manuscript. MAN performed real-time PCR. GR designed and carried out microarray experiments, visualized the models regarding gene expression in the haploids and contributed to drafting and revising the manuscript. LN contributed the ESP gametoclones and provided plant materials to the international collaborators, as well as contributions to the manuscript. PO contributed one of the gametoclones (FRA), contributed to the design of the project, as well as to the manuscript. MT contributed to the design of the work and collaborated in the drafting and revising of the manuscript. FG contributed to the design and coordinated the project, on behalf of the International Citrus Genome Consortium, drafted and revised the manuscript, and is corresponding author. All authors read and approved the manuscript.

## Supplementary Material

Additional file 1: Figure S1 Two homozygous plants of *Citrus clementina* Hort. ex Tan., cv. Nules from France (FRA) and Italy (ITA). The third, from Spain (ESP), was described in Aleza et al. [22]. **Figure S2.** Flow cytometry analyses of DNA content for the plants from France (FRA) and Italy (ITA). Click here for file

## Requesting deposition of data

The array data have been submitted to ArrayExpress. Experiment name: Molecular Characterization of Three Gametoclones of Citrus clementine. ArrayExpress accession: E-MEXP-3926. Specified release date: 2104-01-10.
