# Supplementary material for: Cytological and molecular characterization of three gametoclones of Citrus clementina
Source: BMC Plant Biol. 2013 Sep 10;13:129. doi: 10.1186/1471-2229-13-129 (PMC3847870; doi:10.1186/1471-2229-13-129)
Supplement: Additional file 1: Figure S1 — Two homozygous plants of Citrus clementina Hort. ex Tan., cv. Nules from France (FRA) and Italy (ITA). The third, from Spain (ESP), was described in Aleza et al. [22]. Figure S2. Flow cytometry analyses of DNA content for the plants from France (FRA) and Italy (ITA). [file 1471-2229-13-129-S1.pptx]

## Slide 1
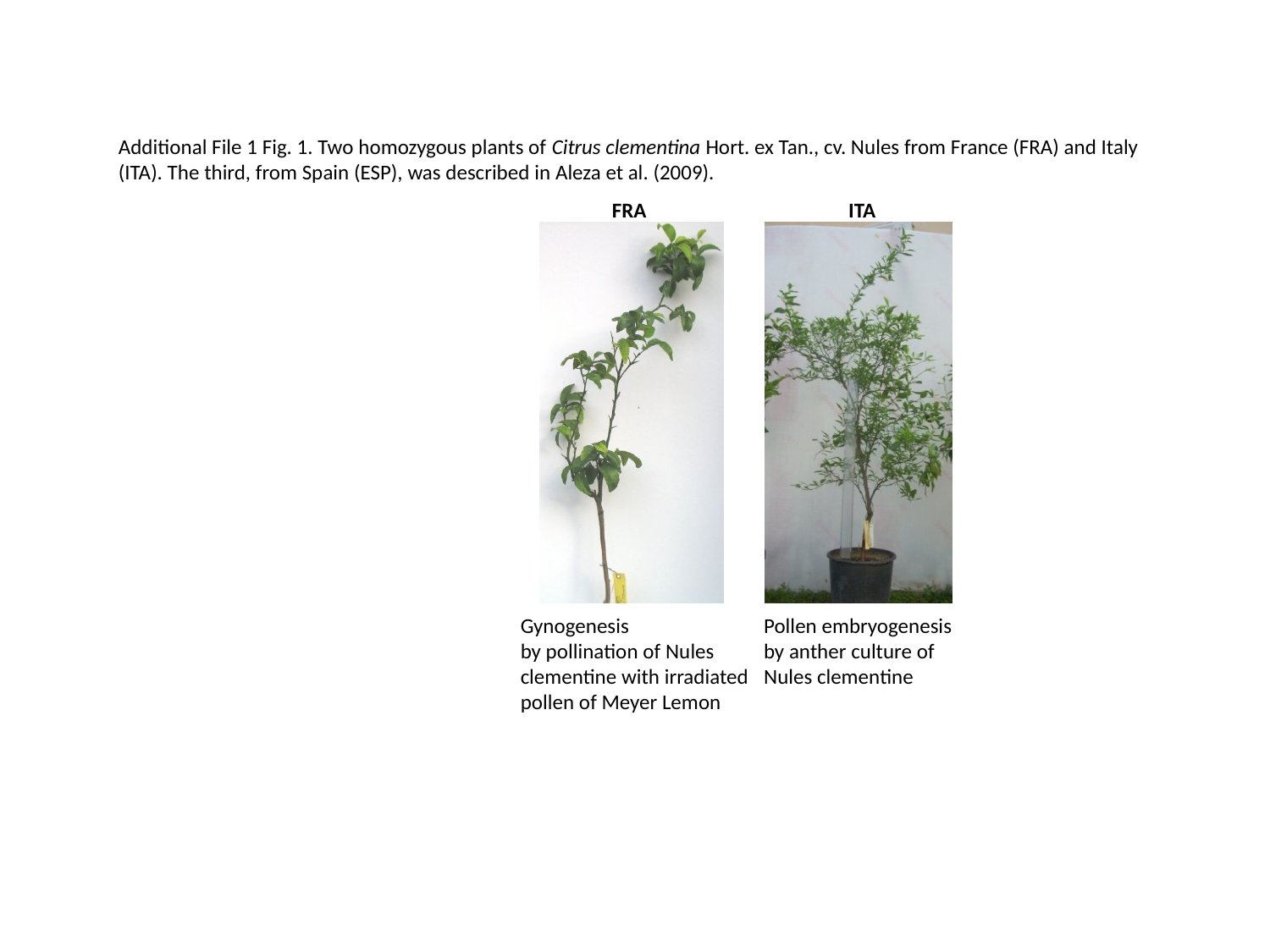

Additional File 1 Fig. 1. Two homozygous plants of Citrus clementina Hort. ex Tan., cv. Nules from France (FRA) and Italy (ITA). The third, from Spain (ESP), was described in Aleza et al. (2009).
FRA
ITA
Gynogenesis
by pollination of Nules clementine with irradiated pollen of Meyer Lemon
Pollen embryogenesis
by anther culture of Nules clementine

## Slide 2
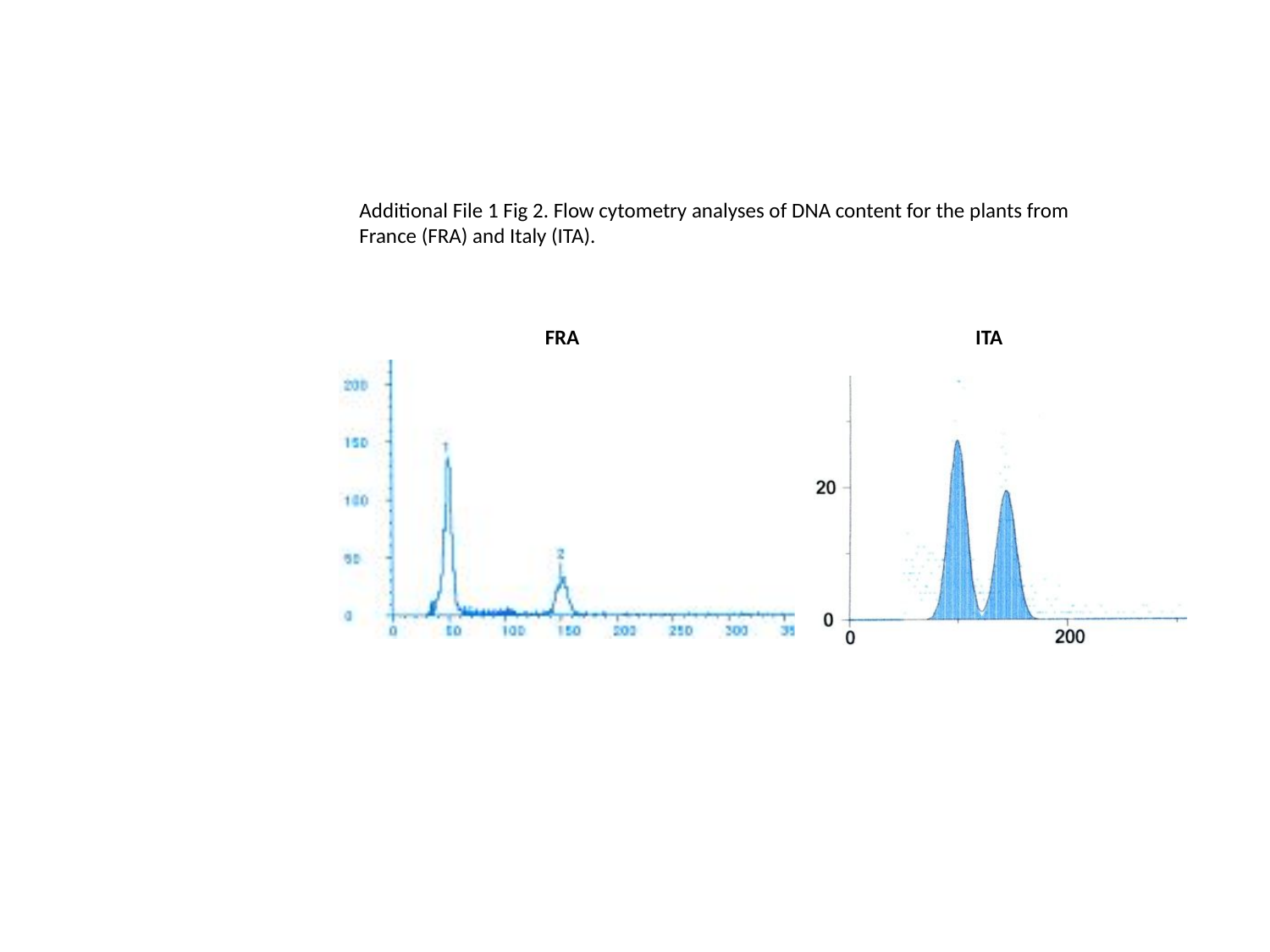

Additional File 1 Fig 2. Flow cytometry analyses of DNA content for the plants from France (FRA) and Italy (ITA).
FRA
ITA
